# Contributions of Age-Related and Audibility-Related Deficits to Aided Consonant Identification in Presbycusis: A Causal-Inference Analysis

**DOI:** 10.3389/fnagi.2021.640522

**Published:** 2021-03-01

**Authors:** Léo Varnet, Agnès C. Léger, Sophie Boucher, Crystel Bonnet, Christine Petit, Christian Lorenzi

**Affiliations:** ^1^Laboratoire des Systèmes Perceptifs, UMR CNRS 8248, Département d'Études Cognitives, École normale supérieure, Université Paris Sciences & Lettres, Paris, France; ^2^Manchester Centre for Audiology and Deafness, Division of Human Communication, Development & Hearing, School of Health Sciences, Faculty of Biology, Medicine and Health, Manchester Academic Health Science Centre, University of Manchester, Manchester, United Kingdom; ^3^Complexité du Vivant, Sorbonne Universités, Université Pierre et Marie Curie, Université Paris VI, Paris, France; ^4^Institut de l'Audition, Institut Pasteur, INSERM, Paris, France; ^5^Centre Hospitalier Universitaire d'Angers, Angers, France; ^6^Collège de France, Paris, France

**Keywords:** sensorineural hearing loss, phoneme identification, aging, presbycusis, audibility deficit, suprathreshold auditory deficits, causal inference

## Abstract

The decline of speech intelligibility in presbycusis can be regarded as resulting from the combined contribution of two main groups of factors: (1) audibility-related factors and (2) age-related factors. In particular, there is now an abundant scientific literature on the crucial role of suprathreshold auditory abilities and cognitive functions, which have been found to decline with age even in the absence of audiometric hearing loss. However, researchers investigating the direct effect of aging in presbycusis have to deal with the methodological issue that age and peripheral hearing loss covary to a large extent. In the present study, we analyzed a dataset of consonant-identification scores measured in quiet and in noise for a large cohort (*n* = 459, age = 42–92) of hearing-impaired (HI) and normal-hearing (NH) listeners. HI listeners were provided with a frequency-dependent amplification adjusted to their audiometric profile. Their scores in the two conditions were predicted from their pure-tone average (PTA) and age, as well as from their Extended Speech Intelligibility Index (ESII), a measure of the impact of audibility loss on speech intelligibility. We relied on a causal-inference approach combined with Bayesian modeling to disentangle the direct causal effects of age and audibility on intelligibility from the indirect effect of age on hearing loss. The analysis revealed that the direct effect of PTA on HI intelligibility scores was 5 times higher than the effect of age. This overwhelming effect of PTA was not due to a residual audibility loss despite amplification, as confirmed by a ESII-based model. More plausibly, the marginal role of age could be a consequence of the relatively little cognitively-demanding task used in this study. Furthermore, the amount of variance in intelligibility scores was smaller for NH than HI listeners, even after accounting for age and audibility, reflecting the presence of additional suprathreshold deficits in the latter group. Although the non-sense-syllable materials and the particular amplification settings used in this study potentially restrict the generalization of the findings, we think that these promising results call for a wider use of causal-inference analysis in audiology, e.g., as a way to disentangle the influence of the various cognitive factors and suprathreshold deficits associated to presbycusis.

## 1. Introduction

According to the most recent estimates (Haeusler et al., 2014; Action on Hearing Loss, 2015; World Health Organization, 2018), about 20% of the population of high-income countries have some degree of hearing loss, and 7–10% have a loss severe enough to create problems in everyday life. Amongst these problems, hearing-impaired (HI) listeners experience difficulties with speech comprehension in natural, noisy, settings. In a society where oral communication is ubiquitous, such degradation in the ability to understand speech can in turn yield difficulties at work, social isolation, and eventually depression or dementia (Gopinath et al., 2012; Amieva et al., 2018).

Sensorineural hearing loss can happen at any age, with any degree of severity. The large majority of cases are however due to late onset forms, mainly presbycusis, the progressive degradation of hearing with age. As estimated by Plomp (1978), the percentage of the U.S. population with problems in perceiving speech approximately doubles with every decade in age. Disabling hearing loss affects one-third of the population over 65, and almost half of all people over the age of 70 (Action on Hearing Loss, 2015; World Health Organization, 2018). Hearing loss is therefore considered a growing public health problem in aging countries.

**Figure 1:**
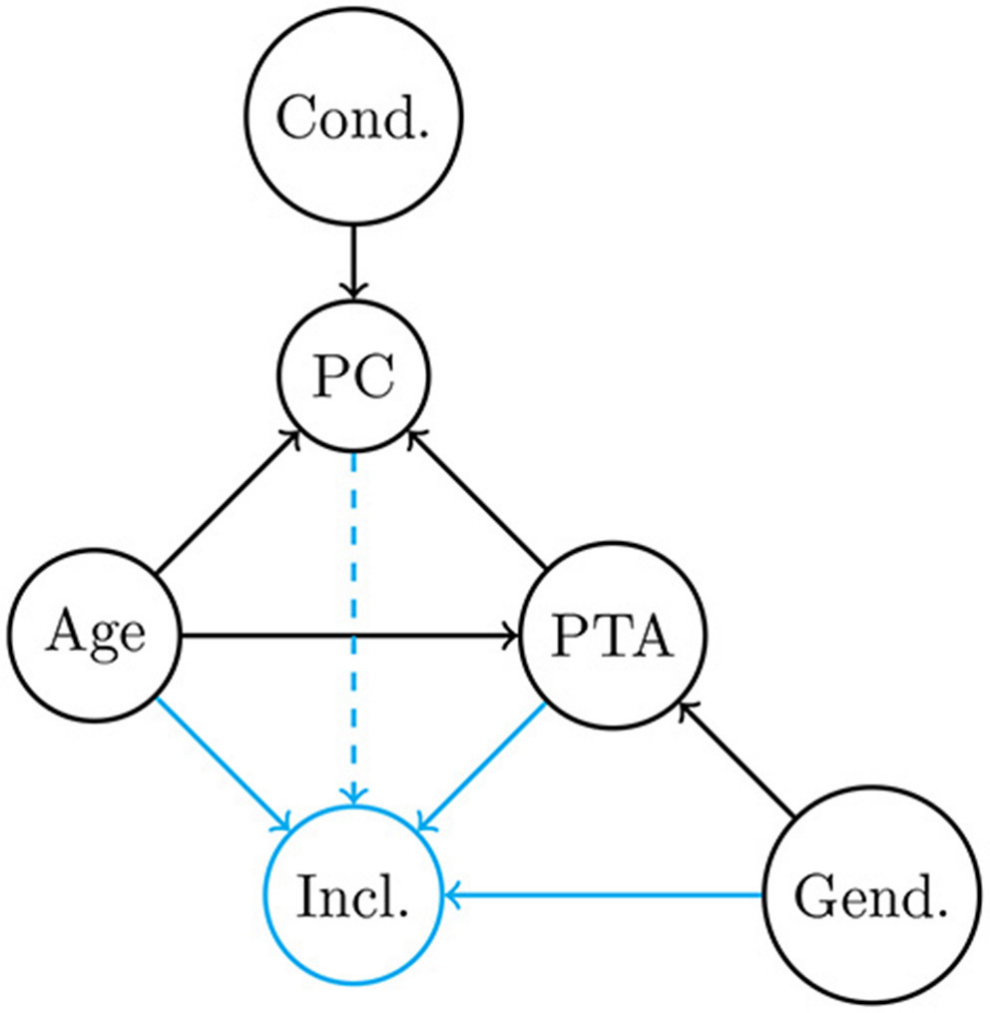
Directed Acyclic Graph (DAG) describing the hypothesized qualitative causal relationships among variables observed in this study (black circles): Age, PTA, Gender, Condition and percentage of correct answers. The blue circle symbolizes the collider “inclusion into the analysis.” Each arrow represents a causal influence between two variables. Black arrows are general dependencies, whereas blue arrows are specific to the setting of this study. The dashed arrow correspond to missing data, which influence is assumed to be negligible (see section 2.4).

Over the last decades, there has been a vigorous debate regarding the respective contributions of factors age and audibility (among others) to the impaired speech intelligibility demonstrated by most presbycusic people. In the next paragraphs we will detail the main causal paths between these factors, which are also summarized as black arrows in Figure 1.

### 1.1. Age-Related and Audibility-Related Deficits in Presbycusis

One of the main characteristics of presbycusis is a gradual deficit in absolute auditory sensitivity. In the clinic, audibility loss is primarily diagnosed using the pure-tone audiogram, a measure of detection thresholds as a function of frequency. It can then be summarized into a single value, the Pure-Tone Average (PTA), corresponding to the mean thresholds over a frequency range relevant for speech comprehension (for example, 0.5, 1, 2, and 4 kHz). The wide use of PTA as a general indicator of hearing loss (e.g., for participant inclusion in audiology studies) can be explained by the simplicity of this measure and the fact that threshold elevation is a robust predictor for unaided speech comprehension in HI people. In correlational studies, audiometric sensitivity typically explains 50–75% of the variance in intelligibility measures across listeners in various tasks^1^ (Festen and Plomp, 1983; van Rooij and Plomp, 1990; George et al., 2007; Houtgast and Festen, 2008; Sheft et al., 2012; Humes, 2013; Maeda et al., 2018). For instance, Humes (2007) reports a strong dependency between the (high-frequency) PTA of 24 unaided HI listeners and their word-recognition scores measured in quiet and against a steady speech-shaped noise (both *R*^2^ > 65%). Indeed, as larger portions of the speech signal become inaudible, its linguistic content is harder to retrieve.

In this context, the main purpose of hearing aids is to counteract the effects of audibility loss by providing a frequency-dependent amplification. When the incoming signal is made audible again, intelligibility generally improves—although rarely up to the normal-hearing (NH) level and with a large variability in the objective and subjective benefit achieved by different individuals (see, e.g., McCormack and Fortnum, 2013; Meister et al., 2015). As a matter of fact, compared to the range of explained variance for unaided HI listeners reported in the above paragraph, HI participants provided with real or simulated hearing aids show a much weaker dependency between PTA and intelligibility scores (*R*^2^ ~ 15–30%, Bernstein et al., 2016; Heinrich et al., 2016; Lopez-Poveda et al., 2017), reflecting the fact that the impact of audibility loss has been somewhat alleviated by amplification. In the best case scenario, the audiogram no longer accounts for a significant proportion of variance in their performances. In the above-mentioned study conducted by Humes, for example, the explained variance for monosyllabic word recognition scores in quiet and in noise fell to < 5% when the stimuli were spectrally shaped prior to presentation to simulate the effect of a hearing aid. However, in general, a residual correlation is still observed between PTA and performance, especially in the most demanding listening situations (up to ~ 50% of explained variance in a spatialized sentence comprehension task using two simultaneous linguistic and non-linguistic maskers; Nuesse et al., 2018).

This remaining portion of explained variance is usually interpreted as evidence that amplification cannot fully eliminate the manifold deficits associated with hearing loss. In practice, even individually-fitted state-of-the-art hearing aids are unable to restore normal speech perception for two reasons: the audibility compensation provided is only partial (even introducing additional distortions in some cases) and additional suprathreshold auditory deficits come into play (Lesica, 2018). Suprathreshold deficits can result from cochlear hearing loss not compensated for by a hearing aid, that correspond to perceptual distortions along the amplitude, spectral and temporal dimensions. They have been shown to relate to the intelligibility of speech, with a particularly deleterious effect on speech-in-noise comprehension (Plomp, 1978; Festen and Plomp, 1983; Houtgast and Festen, 2008; Van Esch and Dreschler, 2015). As a result, differences in speech reception thresholds (SRT) between NH and aided HI listeners are usually more prominent in noise than in quiet. Furthermore, the deterioration in speech intelligibility associated to sensorineural hearing loss is found to be strongly exacerbated with complex background maskers showing temporal or spectro-temporal fluctuations (Lorenzi et al., 2006; Phatak and Grant, 2012). This is usually interpreted as an inability of HI listeners to “listen into the dips,” that is, to make use of the information within the short time windows provided by the fluctuations of the masker. Non-stationary backgrounds are extremely common in daily life, for example when trying to communicate in a crowdy environment full of concurrent voices. Therefore, in many situations where NH listeners can easily follow a conversation, some HI listeners will not understand anything at all. In the laboratory, these masking effects are explored systematically using (notionally) steady speech-shaped (SSN) noise, spectrally and/or temporally modulated SSN noise, a single competing speech source, or a multi-talker babble (e.g., Festen and Plomp, 1990; Lorenzi et al., 2006; George et al., 2007; Léger et al., 2012b,c, 2014; Phatak and Grant, 2012; Meister et al., 2013; Füllgrabe et al., 2014; Van Esch and Dreschler, 2015). As an example, the speech-perception test developed by Gnansia et al. (2009) (“Intellitest”) and used in the present study is a fast vocal audiometry procedure designed for the clinic (Léger et al., 2012a,c; Gnansia et al., 2014). It is based on a consonant-identification task administrated in quiet or against a spectrotemporally modulated SSN, using French material. Finally, suprathreshold auditory deficits also include a higher-level component, corresponding to a reduced processing efficiency, that is, suboptimal encoding and use of sensory information by the central auditory system (Varnet et al., 2019; Venezia et al., 2019).

Elderly people often experience difficulty understanding speech, especially in demanding conditions, although there are large interindividual differences in speech recognition performance among older adults. Considered in isolation, age explains ~ 10–30% of variance in SRT in various tasks for groups of aided or unaided HI listeners (George et al., 2007; Van Esch and Dreschler, 2015; Bernstein et al., 2016; Lopez-Poveda et al., 2017; Tognola et al., 2019). This indicates that there is a link between age and speech recognition in noise, of lesser importance than the predictive effect of hearing loss mentioned earlier. As age-related changes affect both sensory and cognitive processes (Lee, 2015; Jayakody et al., 2018), the negative influence of age on intelligibility is usually explained as a combination of two separate components: (1) a primary effect of age through the elevation of audiometric thersholds and (2) a secondary effect of age through the decline of cognitive or suprathreshold abilities^2^. These effects will be detailed in the following paragraphs.

The most visible effect of age on audition is a progressive decline in auditory sensitivity, due to age-related changes in the cochlea, detected as an increase in hearing thresholds and PTA. Presbycusis typically occurs symmetrically in both ears, beginning with high frequency loss and progressing toward low-frequency sounds (AFNOR, 2017). Furthermore, presbycusis is associated to a higher rate of decline in men than in women, and to a two-fold increase in prevalence (AFNOR, 2017; Jayakody et al., 2018). Hearing sensitivity decline is thought to be one of the main causes for speech understanding deterioration in quiet settings, were sound levels are usually lower and therefore closer to absolute thresholds (Plomp, 1978).

It is tempting to conclude from these data that in the absence of hearing loss there should be no age-related changes in speech perception. This is not the case, however, as elderly with absolute threshold within the NH range also experience difficulties with speech perception, especially in the most demanding listening conditions (Sheft et al., 2012; Meister et al., 2013; Füllgrabe et al., 2014; Schoof and Rosen, 2014; Profant et al., 2019). Similarly, when the effect of PTA is statistically partialed out in a linear regression, age appears as a significant predictor of SRT for aided (Bernstein et al., 2016; Lopez-Poveda et al., 2017; Tognola et al., 2019) and unaided (Van Esch and Dreschler, 2015) HI listeners, although the benefit in terms of additional explained variance is usually limited (Δ*R*^2^ ~ 5%). Also interesting in this respect is a study by Humes et al. measuring sentence-in-noise recognition in older adults provided with an amplification ensuring optimal audibility up to 4 kHz. When doing so, aided speech perception was determined primarily by cognitive and higher-level auditory processes with a significant, but small, contribution from PTA (Humes et al., 2013). The above observations suggest the existence a secondary causal paths linking aging to poorer speech perception. In addition to their elevated absolute thresholds, older adults demonstrate deficits in various suprathreshold auditory or cognitive processes compared to their younger peers. Because these processes are crucial for robust speech perception, researchers have proposed that their decline over time may percolate to speech intelligibility. Two particular factors have been recently given particular attention: auditory temporal processing and cognitive abilities (Houtgast and Festen, 2008; Füllgrabe et al., 2014).

There is now an abundant scientific literature on the crucial importance of temporal cues information in speech sounds (Plomp, 1983; Rosen, 1992; Edwards and Chang, 2013; Ding et al., 2017; Varnet et al., 2017) which form the basis of several models mimicking human speech recognition (Houtgast et al., 2002; Heinz and Swaminathan, 2009; Shamma and Lorenzi, 2013), and it is known that alteration of these cues strongly impairs intelligibility (Ardoint and Lorenzi, 2010; Ardoint et al., 2011). Given that sensitivity to and efficient processing of temporal cues (measured with amplitude- or frequency-modulation detection tasks, gap-detection tasks, or more complex streaming tasks) decline with age (Pichora-Fuller and Souza, 2003; Jayakody et al., 2018), even in the absence of audiometric hearing loss (Füllgrabe et al., 2014; Wallaert et al., 2016; Holmes and Griffiths, 2019; Profant et al., 2019), it seems reasonable to assume that they may be at cause for the difficulties with speech understanding left unexplained by PTA alone. Indeed, both audibility deficits and temporal processing deficits are identified as significant factors in a stepwise regression model predicting aided SRT in noise (Lopez-Poveda et al., 2017). Furthermore, Bernstein et al. (2016) demonstrated that, when factors PTA and auditory temporal processing skills (as measured by spectrotemporal modulation sensitivity) are entered together in a statistical model, the age factor becomes non-significant, suggesting that the effect of age on speech intelligibility could be in fact mediated by the suprathreshold auditory deficits.

Another group of factors which has been investigated for its potential role in the age-related decline in speech intelligibility is cognitive functions. Aging is usually associated with the decrease in attention, working memory, language processing, decision making, executive functions and reasoning. As these abilities are involved in processing, selecting, storing, and recovering information from speech sounds, their impairment generally impact speech-in-noise comprehension (Humes et al., 2012; Lee, 2015; Jayakody et al., 2018), as confirmed by correlational studies conducted on NH or HI older individuals (Pichora-Fuller and Souza, 2003; George et al., 2007; Heinrich et al., 2016; Rönnberg et al., 2016; Tognola et al., 2019; for an overview, see Akeroyd, 2008; Humes et al., 2012). Among the battery of tests usually administered to measure cognitive functions, those related to working memory generally show the strongest predictive effect (Akeroyd, 2008; Meister et al., 2013; Souza et al., 2015). Finally, the comparison of *R*^2^ values for nested linear models confirms that, for complex maskers, a portion of the variance in SRT explained by age can in fact be attributed to cognition (Meister et al., 2013).

Although this body of evidence points toward an overall role of suprathreshold auditory processes and cognition in the speech-in-noise comprehension deficit in the elderly, it is worth noting that the precise mechanisms involved are still unclear. In particular, no single cognitive function appeared as significant across all studies (Akeroyd, 2008) and several researchers were unable to find a significant link between speech recognition in noise and the cognitive abilities they measured in their pool of participants (Schoof and Rosen, 2014; Meister et al., 2015; Lopez-Poveda et al., 2017; Nuesse et al., 2018). Similarly, studies are not always consistent on the role of auditory temporal processing skills on aging speech perception. Several authors have found no difference between old and young adults on tests meant to tackle auditory temporal processing functions (Schoof and Rosen, 2014) or no contribution of these interindividual differences to the performance in speech recognition (Takahashi and Bacon, 1992). More generally, it is thought that the relative role of audibility vs. suprathreshold- or cognitive factors (and therefore the measured effect of age) may vary with the complexity of the task (Akeroyd, 2008; Schoof and Rosen, 2014; Van Esch and Dreschler, 2015; Heinrich et al., 2016). An appealing explanation is that higher-level processes become a critical factor only beyond a certain degree of hearing loss (Füllgrabe and Rosen, 2016) or acoustic degradation (George et al., 2007; Souza et al., 2015), that is, only when the input needs to be supplemented by top-down processes.

### 1.2. Causal Inference Analysis

The above-described set of causal relationships between variables PTA, age, listening condition (cond) and speech intelligibility (PC) is summarized as black arrows in the directed acyclic graph (DAG) in Figure 1. As it is clear from this diagram, age is connected to PC via two different causal paths: a direct one (age → PC) and an indirect one (age → PTA → PC). In other words, researchers investigating aging effects in sensorineural hearing loss have to deal with the methodological issue that factors age and peripheral hearing loss covary to a large extent (Martin et al., 1991). One solution for studying the specific effects of variables that are impossible to manipulate experimentally is statistical control, combined with a careful examination of the possible dependencies in the data to avoid introducing spurious associations by controlling for a collider. Expanding on the rationale behind the statistical control of third variables, causal inference analysis offers a principled approach to draw valid causal effect estimates on the basis of observational data (Rohrer, 2018). This can be carried out through a relatively simple graph analysis of the underlying DAG based on the so-called “back-door path” criterion proposed by Pearl (Pearl, 1995, 2000; Textor et al., 2016). The principle behind a causal inference analysis is to rely on an assumed set of causal relationships (summarized in the DAG) to design statistical models for the purpose of estimating the strength of each causal effect of interest. This approach therefore allows us to formally disentangle the direct causal effect between two observed variables from potential indirect confounding effects due to the existence of colliders.

In the present study, we will rely on causal-inference analysis to quantify the separate contributions of audibility- and age-related deficits to speech-intelligibility deficits in quiet and in noise. We applied this approach to a set of data collected over the years 2009–2012 as part of a larger project on the genetics of presbycusis (see Boucher et al., 2020). Our aim was to quantify the relative strength of two components of presbycusis, namely the PTA-related and age-related deficits, on phoneme identification measured in quiet and against a modulated noise, on a very large pool of HI participants recruited from 7 different French audiological/ENT clinics.

A potential danger in such statistical analysis is over-fitting the data by including too many predictors into the model. Here, we will rely on a Bayesian model with conservative priors to select the most predictive variables and to shrink inessential weights toward zero (regularization). Furthermore, another advantage of the DAG analysis mentioned above is that it unambiguously identifies a minimal set of control variables to be included in the model. To the best of our knowledge, such a Bayesian causal-inference analysis has never been carried out to measure the direct contribution of age-related and audibility-related deficits to speech intelligibility in presbycusis.

## 2. Materials and Methods

The data described here was collected over the years 2009–2012 as part of a larger project on the genetic factors of presbycusis (see Boucher et al., 2020).

### 2.1. Participants

We studied patients with an onset of presbycusis after the age of 40 years referred to several audioprosthesists between 2003 and 2012 in seven French university hospitals nationwide (in Marseille, Lille, Paris, Clermont, Lyon, Bordeaux, and Toulouse). All listeners were fully informed about the goal of the study and provided written consent before their participation. The inclusion and exclusion criteria were checked with a standardized questionnaire filled in at all the clinical centers, as well as otological and audiometric examinations (see below).

The study was approved by French “Regional Ethics Committees” (CCPPRB Paris Necker and CPP Ile de France II, Promoteur: RBM 03-37, CPP: 03-10-01, DGS: 2003/0494).

### 2.2. Inclusion Criteria

NH and HI listeners were included in the study based on their age and best-ear bone-conduction audiometric thresholds computed over 0.5, 1, 2, and 4 kHz (later referred to as PTA and measured in dB HL) as a function of age for male and female NH and HI listeners.

**Figure 2:**
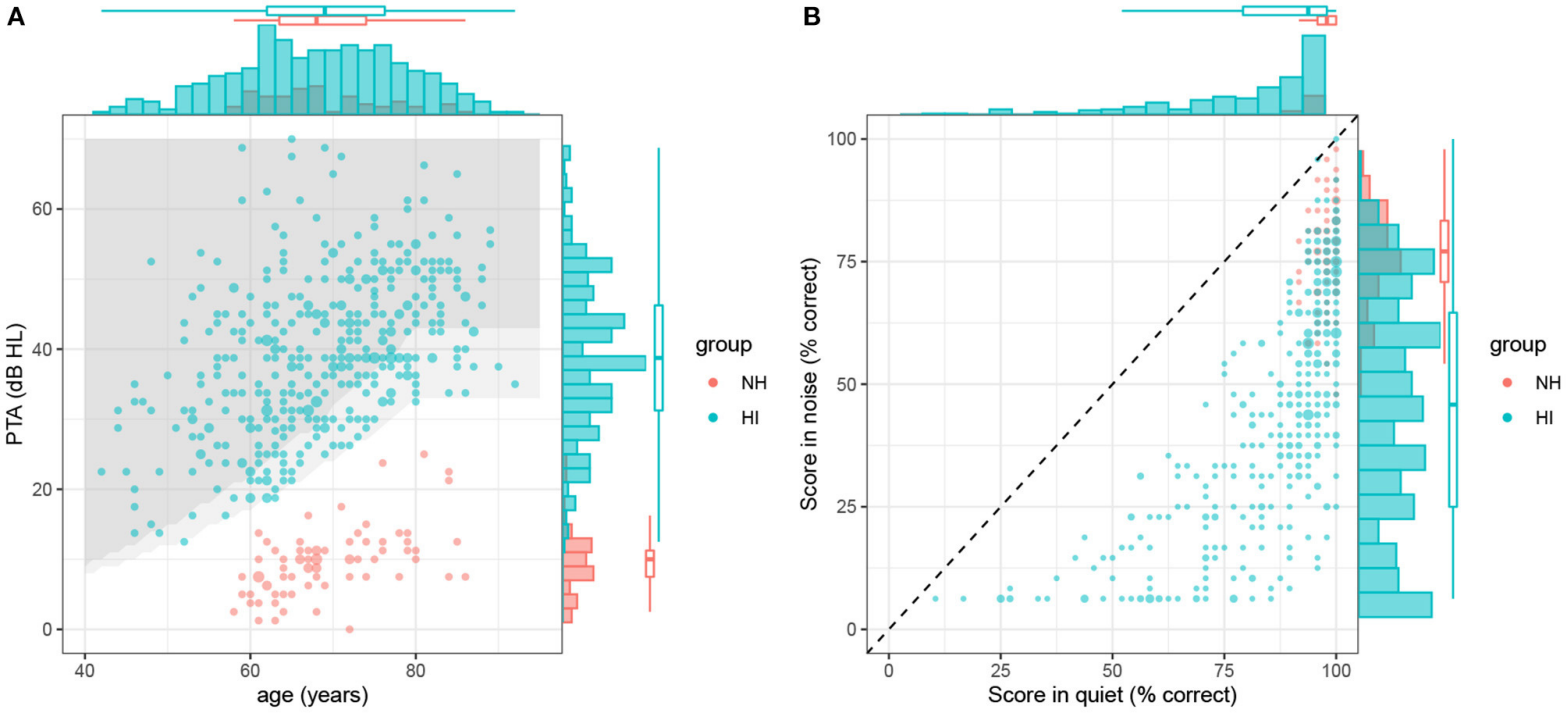
Distribution of the predictors (PTA and age) and outcomes (scores in silence and noise) for all NH (red dots) and HI (blue dots) participants included in the study. **(A)** Joint distribution of PTA and age. The marginal densities for the two variables are indicated with histograms and boxplots. The two gray-shaded areas delineate the PTA-based inclusion criteria for male and female listeners, respectively. **(B)** Identification scores in quiet and noise. The diagonal dotted line shows the identity line (i.e., no effect of masking noise).

HI listeners had to be older than 40 years. In the best ear, the PTA had to be higher than 75% of the French population of the same age and gender (AFNOR, 2017), so as to ensure that our sample is representative of the French population. In addition, audiometric thresholds at 0.5, 1, 2, and 4 kHz had to be lower or equal to 85 dB HL, the audiometric configuration had to be either flat or sloping in the high-frequencies, and the PTA had to be lower or equal to 70 dB HL. The PTA-based inclusion criteria for male and female HI participants are illustrated in Figure 2 (shaded areas), together with the best-ear PTA of the listeners as a function of age for our sample of NH and HI listeners.

NH listeners had to be older than 55 years. This difference in the lower age limit between the two participant groups was set for purely practical reasons related to the recruitment process. In the best ear, bone-conduction audiometric thresholds at 4 kHz had to be less than that of 80% of the French population of the same age and gender (AFNOR, 2017). Furthermore, audiometric thresholds at 0.5, 1 and 2 kHz had to be lower or equal to thresholds at 4 kHz.

For both groups, the difference in audiometric thresholds in the two ears had to be lower than 20 dB HL at each frequency tested. A proportion of participants also self-reported tinnitus. Exclusion criteria for the HI group included: neurological disease at the origin of the hearing loss, syndromic deafness, diabetes mellitus treated by oral antidiabetics or insulin, bilateral chronic otitis, bilateral cholesteatoma, vestibular schwannoma, noise-induced hearing loss, ischemic cardiovascular disease.

### 2.3. Description of the Sample

**Figure 3:**
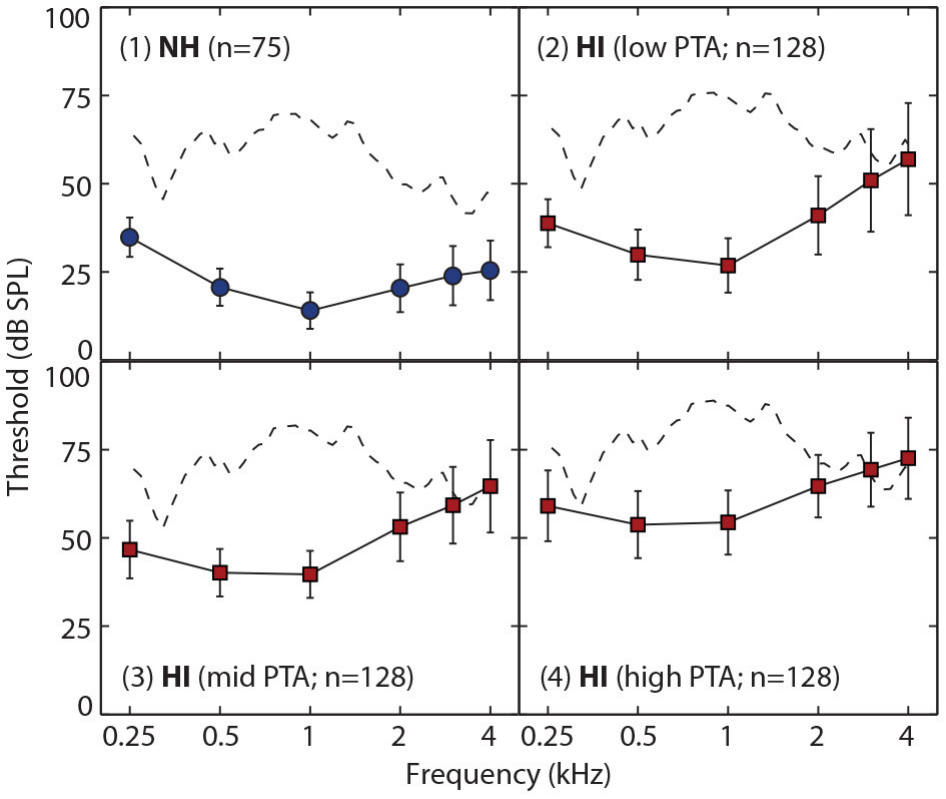
Average bone-conduction audiometric threshold (converted to dB SPL [Sound Pressure Level]) as a function of frequency (in kHz). Thresholds for NH listeners are reported in the top left panel, using blue circles. Thresholds for HI listeners are reported in the 3 remaining panels, using red squares. For illustration purposes, HI listeners were split into 3 groups of 128 listeners on the basis of their PTA: listeners were ranked by increasing order of PTA, and the 128 first listeners were attributed to the group “low PTA,” the following 128 to the group “mid PTA,” and the remaining 128 to the group “high PTA” (note that the PTA was computed over 4 frequencies only). The error bars show the standard deviation about the mean. In each panel, the dotted line shows the long-term spectrum of the aggregated speech signals. These speech signals are shown at a level (in dB SPL) that corresponds to what would have been presented to a listener with the same audiogram as the average reported in that panel. This illustrates how the amplification and spectral shaping of the speech were implemented as a function of audiometric thresholds.

In total, 459 listeners were included, consisting in 75 NH listeners (45% female) and 384 HI listeners (63% female). For each group, Figure 2A shows the joint (scatterplots) and marginal (histograms and boxplots) distributions of age and PTAs, overlaid with the inclusion criteria for HI participants. Average audiometric thresholds are also reported in Figure 3 for NH listeners and 3 sub-groups of HI listeners.

HI listeners (*n* = 384) were 42–92 years old, with a mean of 69 years (median = 69 years, SD = 10 years). PTAs ranged between 13 and 70 dB HL, with a mean of 39 dB HL (median = 39 dB HL, SD = 11 dB HL). Losses ranged from near-normal to moderate-to-severe. Note that 35% of HI listeners reported tinnitus in the tested ear (136 out of 384).

NH listeners (*n* = 75) were 58–86 years old, with a mean of 69 years (median = 68 years, SD = 7 years). PTAs ranged between 0 and 25 dB HL, with a mean of 10 dB HL (median = 10 dB HL, SD = 5 dB HL). PTAs were generally lower or equal to 20 dB HL at 0.5, 1, 2, and 4 kHz; although for 10 listeners thresholds were between 20 and 35 dB HL at 2 and/or 4 kHz. Note that 33% of listeners reported tinnitus in the tested ear (25 out of 75).

The NH and HI groups were comparable in age, despite different inclusion criteria (the minimal age of inclusion was higher for NH than for HI listeners). Furthermore, the sample of HI listeners reproduces the natural dependency between PTA and age measured in the French population thanks to the joint inclusion criteria between the two factors described above.

### 2.4. Speech Task (Intellitest)

Consonant identification scores were measured using 48 non-sense vowel-consonant-vowel-consonant-vowels (VCVCVs) spoken by a French female talker in silence. The VCVCV stimuli consisted of three recordings of 16 /aCaCa/ utterances (C = /p, t, k, b, d, ɡ, f, s, ʃ, v, z, ʒ, m, n, ʁ, l/). VCVCVs were presented either in silence, or against a spectro-temporally modulated background noise.

Before the modulations were introduced, the background noise was a steady-state speech-shaped noise (SSN) masker whose spectrum matched the average spectrum of the whole set of speech stimuli. To introduce the temporal modulation, the SSN was modulated in amplitude at 8 Hz (sinusoidal modulation, modulation depth of 100%). To introduce the spectral modulation, the SSN was passed through 32 non-overlapping gammatone filters (with center frequencies logarithmically spaced over the range 80 to 8,020 Hz), each with a bandwidth of 1 *ERB*_*N*_, where *ERB*_*N*_ is the equivalent rectangular bandwidth of the auditory filter for young NH listeners (Glasberg and Moore, 1990). The output of one filter out of two was set to zero, giving an alternating pattern of one *ERB*_*N*_ removed and one *ERB*_*N*_ present. The starting phase of both modulations was randomized—in other words, for the spectral modulation, the number (1 or 2) of the lowest auditory filter whose output was set to zero was randomized.

**Figure 4:**
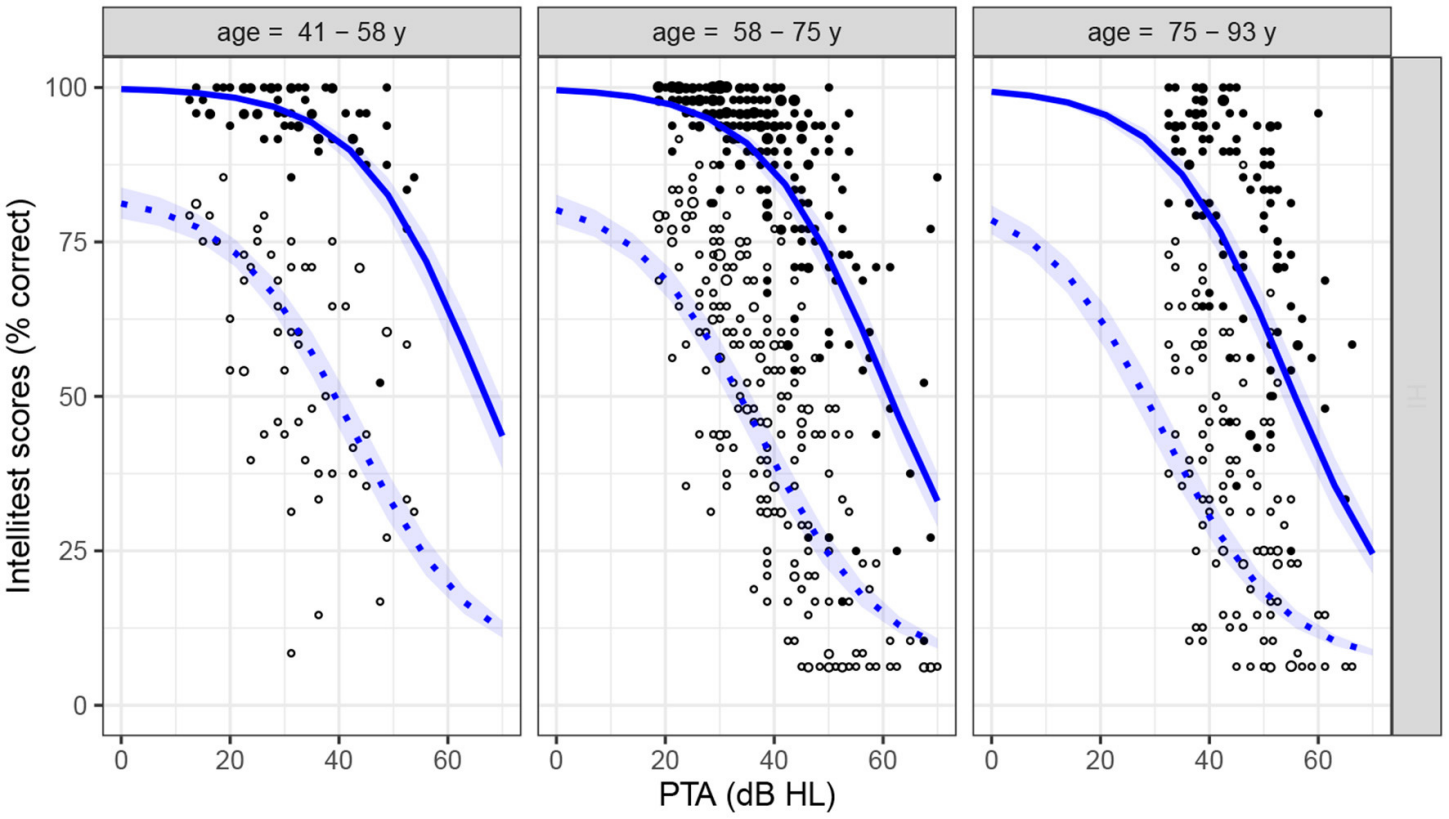
Intellitest scores in quiet- (solid dots) and noise- (open dots) conditions for the HI group, as a function of PTA and age. Age is displayed as a three-level factor for representation purpose only. The two lines show the counterfactual predictions (effect of varying PTA alone) from the PTA-based main-effect model in the two conditions (quiet: continuous lines; noise: dashed lines; shaded area: credible interval). See detailed description in text.

Speech was presented in the best ear for all listeners. The presentation level of the speech before amplification was 65 dB SPL at 1kHz. For HI listeners, the speech was amplified using the Cambridge formula (Moore and Glasberg, 1998):

(1) $$\begin{array}{r} Gain\left(f\right)=HL\left(f\right)\cdot 0.48+Intercept\left(f\right) \end{array}$$

where *HL*(*f*) stands for the audiometric threshold at frequency *f* in dB HL and *Intercept*(*f*) for a frequency-dependent amplification value (see Moore and Glasberg, 1998, Table 1). Examples of the effect of this frequency-dependent amplification on the speech stimuli are presented in Figure 3. The long-term spectrum of the speech signals is shown in four cases: no amplification (Figure 3.1), and with frequency-dependent amplification for 3 levels of hearing loss (Figure 3.2–4). These example audiograms correspond to the average audiogram of NH listeners (Figure 3.1), and the average audiograms of HI listeners categorized in 3 sub-groups (as a function of the severity of their loss; Figure 3.2–4). Note that for HI listeners with the most severe sloping loss (Figure 3.3-4), speech information above 2 kHz might not be audible, despite a larger amplification in the high-frequencies than in the low-frequencies. For all listeners, the noise was presented at a signal-to-noise ratio (SNR) of −4.5 dB. This SNR was chosen during a pilot phase, because it led to an average identification score of 90% for 10 young NH listeners, therefore limiting ceiling effects for the best performers while allowing for a drop in performance for poorer performers.

**Table 1:** Comparison the 5 models described in this study in terms of in-sample and out-of-sample accuracy.

**Model**	***p***	**$\rho_{quiet}^{2}$**	**$\rho_{noise}^{2}$**	**D**	**LOOIC ± SE**	**Δ*LOOIC* ± SE**
PTA-based main-effect model	14	46.8%	53.2%	6302.5	6363.3 ± 232.2	0.0 ± 0.0
PTA-based full model	18	47.7%	59.6%	6286.4	6365.9 ± 235.4	−1.3 ± 10.7
ESII-based full model	18	49.8%	57.1%	6291.7	6375.8 ± 226.5	−6.3 ± 49.8
PTA-only model	13	46.3%	57.3%	6510.4	6567.7 ± 230.9	−102.2 ± 37.4
Age-only model	13	17.0%	25.0%	9039.0	9129.0 ± 374.8	−1382.8 ± 149.7

*Reported for each model are the number of parameters (p), the amount of explained variance in the two conditions (ρ^2^), the in-sample deviance of the fit (D), the estimated out-of-sample deviance LOOIC, and the difference in LOOIC from the best model (ΔLOOIC). Models are ordered by decreasing out-of-sample predictive accuracy (i.e., increasing LOOIC)*.

The Intellitest procedure went as follow. For each background condition (silence and noise), one set of 48 VCVCVs was presented in a random order. During each trial, a single logatome was presented and the listener had to identify the stimulus among 16 alternatives (letter strings corresponding to the non-sense syllables) displayed on a computer screen. Response time was not limited, and no feedback was provided. A testing session lasted 10–20 min, during which the listeners were presented with the 2 background conditions in a random order. Consonant identification scores were measured in percent correct (PC).

Due to the way the data is stored by the Intellitest software, scores lesser than the chance level (i.e., less than 3 correct responses out of 48) were lost and replaced by chance level instead. These chance-level scores were very unevenly distributed across the final dataset: they occurred only in the noise condition and for HI participants with high PTA and/or age. Therefore, this might result in a slight underestimation of the effects of these factors by the models. However, it should be noted that chance-level scores represented a very limited amount of the data (only 33 scores were equal to 3, i.e., 3.7% of the total) and they include a portion of “true” scores. Therefore, the induced bias (dashed arrow in Figure 1) was assumed to be negligible.

### 2.5. Causal Inference Analysis

As a visual representation of the assumed causal structure underlying a given data set, a DAG is a powerful tool to think about the relationships observed between variables. Complemented with the theoretical framework developed by Pearl (Pearl, 1995, 2000), it can be used to (1) deduce which statistical models can provide causal-effect estimates on a set of variables and (2) derive implied conditional independencies for testing the validity of the DAG.

We grounded our analysis on the DAG represented in Figure 1. Black arrows encode general dependencies between variables. Their theoretical and empirical justifications are summarized in the section 1. Blue arrows are specific to the setting of this study, and result from the inclusion criteria described in the section 2. As detailed in the Speech task subsection, the dotted arrow was assumed to be negligible.

The DAG was analyzed using the R package Dagitty (Textor et al., 2016) to identify a minimal sufficient adjustment set for estimating causal effects and to derive testable implications of the DAG. Assuming that the DAG is true, no additional control variable needs to be included in the model in order to retrieve the true causal relationships of Age, PTA, and listening condition on correct identification performance. In particular, controlling for factor gender is possible, but not required, as it is neither a confounder nor a collider in this analysis. On the contrary, in the case of a model where factor PTA or Age is omitted (PTA-only and age-only models, see below), the gender factor needs to be statistically controlled for to block the indirect path Age → Inclusion ← Gender → PTA. For this reason, gender will be included into all models described in this article. Note, however, that the coefficient associated to gender in the model should not be interpreted as a measure of the effect of gender on scores (“Table 2 fallacy,” Westreich and Greenland, 2013).

Due to the limited number of variables considered, only a single relevant conditional independency could be derived from the DAG: PC should be statistically independent of gender, conditional on age and PTA in order to confirm the consistency of the DAG with our data. This statistical property of the data was confirmed by our analysis.

### 2.6. General Model Definition

Apart from the last model which is aimed at comparing the performances of NH and HI participants (see section 2.7), all models were fitted on the HI data only.

The standardized variables age and PTA and the 2-level factors cond (0: quiet; 1: background noise) and gender (0: female, 1: male) were entered as predictors in a logistic regression model. The outcome of the model was *N*_*corr*_, the count of correctly identified consonants across the 48 trials:

(2) $$\begin{array}{r} N_{corr}\sim \;Binomial\left(48,p\right) \end{array}$$

The probability of correct identification *p* is assumed to follow the general form for n-alternatives categorization protocols (see, e.g., Wichmann and Hill, 2001):

(3) $$\begin{array}{r} p=\frac{1}{16}+\left(1-p_{miss}-\frac{1}{16}\right)\cdot p^{\prime } \end{array}$$

with 1/16 being the chance level (selection of 1 consonant amongst 16 possible answers) and *p*_*miss*_ the probability of lapses. These two parameters are reflected in the upper- and lower- bounds of the intelligibility curve being lower than 100% correct and higher than 0% correct, respectively. *p*_*miss*_ was assumed to be equal to 0 in quiet; however, the data from the NH and HI groups clearly indicates that it is positive in the noisy condition.

Finally, *p*′ is described with a standard logistic regression formula. The initial model (“PTA-based main-effect model”) includes only the main effects of PTA, age, cond, and gender:

(4) $$\begin{array}{l} logit\left(p^{\prime }\right)=\gamma_{0,site}+\beta_{PTA}\cdot PTA+\beta_{age}\cdot age+\beta_{cond}\cdot cond\\ \;+\beta_{gender}\cdot gender \end{array}$$

Note that the presence of background noise has two distinct effects on performance in the model: a multiplicative effect through *p*_*miss*_ and a logistic-additive effect through *β*_*cond*_. These two effects are usually investigated separately in the speech-in-noise literature; here, the Bayesian framework allows for their joint estimation.

The difference in scores between testing sites prompts the use of a hierarchical varying-intercept model (Gelman and Hill, 2006) to account for dependencies across and within testing sites. Each site was associated with a specific intercept *γ*_0,*site*_, and the distribution for the 7 intercepts was described with two hyperparameters *β*_0_ and *σ*_0_ representing the mean and standard deviation across sites: $\gamma_{0,site}\sim \;N\left(\beta_{0},\sigma_{0}\right)$. The dispersion of the seven site-specific parameters was associated to a reasonably strong prior *σ*_0_ ~ Half-Normal(0,0.05), enforcing the pooling of information to improve estimate in testing sites with smaller sample sizes. Weakly informative, conservative distributions $\beta_{X}\sim \;N\left(0,1\right)$ were used for all other effect-related parameter and for hyperprior *β*_0_ (recall that all predictors were expressed on a standardized scale). Finally, the distribution of *p*_*miss*_ was estimated within the model, with a weak beta-distribution prior. In practice, the model was implemented with non-centered priors to improve efficiency. The use of conservative prior distributions ensures a form of *L*_2_ regularization on the model weights and can be seen as a Bayesian counterpart to the stepwise regression procedure extensively used in correlational studies (George et al., 2007; Van Esch and Dreschler, 2015; Lopez-Poveda et al., 2017; Holmes and Griffiths, 2019), as it effectively shrinks weights not contributing to prediction toward zero. A visual summary of the hierarchy of priors associated with each parameter in the model is provided in Supplementary Figure 1.

Three variants of the above-described model were also considered: two models with factors PTA or age removed (“age-only model” and “PTA-only model,” respectively), and a full model including all interaction effects between predictors PTA, age, and cond. See section 1 in Supplementary Materials, for more details.

All data were analyzed in R version 3.6.3 (R Core Team, 2020), using the RStan interface to Stan for Bayesian modeling (Carpenter et al., 2017; Stan Development Team, 2020). Seven chains of 7,000 samples each were run independently (3,000 burn-in samples, estimates based on 4,000 samples). Their convergence was monitored through standard summary statistics R-hat and Effective Sample Size, absence of divergent transition, and visual inspection of posterior distributions. Throughout this article, Bayesian estimates will be reported along with their 95% credible intervals, providing an assessment of the reliability of the results. All the codes and data supporting this analysis are openly available on Github at https://github.com/LeoVarnet/Presbycusis_analysis.

### 2.7. ESII

The contribution of audibility to speech identification scores was quantified using the extended speech-intelligibility index (ESII) of (Rhebergen and Versfeld, 2005) and (Rhebergen et al., 2006). The ESII estimates speech intelligibility based on the target and masker spectra and on the individual audiometric thresholds. Unlike the original SII, the ESII is computed separately in successive short time frames, and then averaged across time, which makes it suitable for predicting speech intelligibility in modulated maskers (Rhebergen et al., 2006). Note that to account for the temporal effects that affect speech intelligibility in non-stationary backgrounds, the ESII is not entirely based on audibility estimates, but also includes a forward masking function (in dB as a function of time). The forward-masking function is linear (for time on a logarithmic axis, see Figure 6 in Rhebergen et al., 2006) and its duration is always equal, regardless of the absolute thresholds. As a consequence, the slope of the forward-masking function varies with the absolute thresholds, or in other words, the decay of forward-masking (in dB) is faster for NH listeners than for HI listeners. Therefore, one might argue that the ESII models speech intelligibility on the basis of both audibility and some supra-threshold deficits (namely, forward masking).

One potential limitation to the above statement is that the ESII calculation relies on the choice of a particular frequency-weighting function, specific to the type of speech materials and test considered, and which combines the information about the audibility in each band into a single metric (Rhebergen and Versfeld, 2005; Rhebergen et al., 2010). As no frequency-weighting function has been yet developed and validated for the Intellitest material, we decided to use the closest available option: the speech in noise (SPIN) test, which involves a monosyllabic-words-in-noise recognition task. A major difference with the Intellitest is that the target monosyllables are presented within a sentence. Therefore, the obtained ESII values may not be accurate as they rely on the incorrect assumption that a certain amount of semantic information is available to the listeners, a situation which may potentially change the contribution of each spectral band to speech intelligibility. However, the present analysis carried with an alternative frequency-weighting function (NNS option) yielded essentially the same results.

ESII values were computed on an individual basis for NH and HI listeners in each listening condition, using the actual spectra of the speech and noise stimuli from the Intellitest materials and the presentation levels and amplification corresponding to each participant. These individual values were then used as a predictor for the ESII-based statistical models. A first model (“ESII-based full model”) with a similar structure as the PTA-based full model described above was fitted on the HI data only and compared to the PTA-based models. Yet, another advantage of ESII over PTA is that it provides a common basis for comparing aided HI listeners with unaided NH listeners. Therefore, as a last step, a second ESII model was fitted on the full dataset. The aim was not to compare this model with the ones previously described, as they are fit to different numbers of observations, but to try to compare the variability within the NH and HI groups. For this purpose, a two-level *group* factor was added to the model, as well as its interactions with age, ESII and cond. In order to keep the sampling algorithm tractable, second- and third-level interactions with group were not considered. Furthermore, because of the very limited range of ESII within the NH group and the quiet condition, the interaction effect *group* * *ESII* was not considered either. The squared prediction error (on the log-odds scale) was then compared between the two groups, for the two conditions.

### 2.8. Model Comparison

Models fitted on HI data only were compared on the basis of their out-of-sample predictive accuracy and their parameter posterior distributions—two complementary approaches, aimed at understanding how adding predictors into the model changes predictions and estimates. The use of out-of-sample (rather than in-sample) metrics is important to avoid overfitting, especially when comparing between models with different numbers of parameters as it is the case here.

The ability of the model to generalize predictions to a new dataset was assessed with PSIS-LOO, an estimator of the leave-one-out (LOO) cross-validation predictive accuracy (Vehtari et al., 2017). This value is expressed on a deviance scale as the leave-one-out information criterion (LOOIC). For every LOOIC, Pareto k diagnostic values were checked for reliability (all k above 0.7, see Vehtari et al., 2017). The Watanabe–Akaike information criterion (WAIC) yielded essentially the same results and will not be reported here.

Although model selection was performed on the basis of the out-of-sample predictive accuracy metrics LOOIC only, the percentage of explained variance of each model (predictive accuracy on the training dataset) were also evaluated and reported in order to relate our results to previous studies. Arguably, the counterpart for *R*^2^ in the case of a logistic regression model is *ρ*^2^, the percentage of explained variance on the linear log-odds scale underlying the probability of correct response (DeMaris, 2016). In order to improve comparability with previous speech-in-noise studies, two *ρ*^2^ were calculated separately on the quiet and noise conditions, although the model was fitted simultaneously on all available data.

In-sample and out-of-sample predictive accuracy metrics were compiled in a single table (Table 1) to facilitate comparisons across models.

## 3. Results

The Intellitest scores in quiet and in noise for each participant are shown in Figure 2B. All data points but two deviated from the diagonal dotted line, confirming the deleterious effect of the modulated noise masker on intelligibility. The HI group obtained scores ranging from 10.4 to 100% in quiet (mean = 85.8%) and from chance (6.25%) to 100% in noise (mean = 45.5%). For the NH group, scores ranged from 91.7 to 100% in quiet (mean = 97.6%) and from 47.9 to 97.91% in noise (mean = 97.6%). Scores were markedly different between sites. For instance, participants in Bordeaux reached higher scores in noise (mean = 55.0%, sd = 21.6%) than participants in Toulouse (mean = 28.8%, sd = 22.0%).

Figure 4 plots the data for the HI group only (Intellitest scores in quiet [solid dots] and in noise [open dots]) as a function of PTA and age, making apparent the dependencies between the three variables. A similar representation of the data from each testing site can be found in the Supplementary Materials.

The influence of PTA and age on scores in quiet and in noise was first evaluated with a varying-intercept hierarchical model including only 4 predictors: PTA, age, condition, and gender (“PTA-based main-effect model”). The posterior distribution for the model parameters revealed strong negative effects of noise (*β*_*cond*_ centered around −2.07, *CI*_95%_ = [−2.17, −1.97], 40 SD away from zero) and PTA (mean = −1.36, *CI*_95%_ = [−1.43, −1.30], 41 SD away from zero), and a smaller effect of age (mean = −0.27, *CI*_95%_ = [−0.31, −0.23], 14 SD away from zero). Only two testing-site-specific intercepts robustly differed from the mean of the group: Paris (mean distance from group = 0.61, *CI*_95%_ = [0.42, 0.81]) and Toulouse (mean distance from group = −1.07, *CI*_95%_ = [−1.35, −0.80]). The gender factor, which was included as a statistical control, was not associated to a strong coefficient (mean = −0.03, *CI*_95%_ = [−0.10, 0.03], 0.9 SD away from zero), consistent with the conditional independencies implied by the DAG (see section 1.2). Finally, the model yielded a reliable estimate of *p*_*miss*_ to 17.2 % (*CI*_95%_ = [14.1, 19.9%]). Posterior distributions for all parameters in the model are shown in Supplementary Figure 2.

As the conservative priors in the model effectively implement a form of parameter selection by shrinking all weights toward zero, the above results suggest that PTA and age have a complementary explanatory value (i.e., that individual variations in age predict a portion of the variance in scores left unexplained by PTA), which would indicate a causal effect of age not mediated by PTA. Furthermore, the model provides a reliable estimate of the relative strength of the two effects : the effect of PTA was 5.0 times higher than the effect of age (*CI*_95%_ = [4.3, 5.9]). The predictive accuracy of the model was assessed both in terms of goodness-of-fit ($\rho_{quiet}^{2}=46.8\%$ and $\rho_{noise}^{2}=53.2\%$ of variance explained, training deviance = 6302.5) and estimated out-of-sample deviance (6363.3 ± 232.2 SE). The counterfactual predictions according to the main-effect model (mean Intellitest scores across- and within- sites as a function of PTA, all other parameters being fixed) are displayed in Figure 4 and Supplementary Figure 4^3^.

In order to confirm the complementary roles of PTA and age, two nested models were fitted and compared with the above model by subtracting factor PTA or age from equation 4, respectively. As expected, the prediction accuracy for the nested models was lower (age-only model: $\rho_{quiet}^{2}=17.0\%$, $\rho_{noise}^{2}=25.0\%$, training deviance = 9039.0, out-of-sample deviance = 9129.0 ± 374.8 SE; PTA-only model: $\rho_{quiet}^{2}=46.3\%$, $\rho_{noise}^{2}=57.3\%$, training deviance = 6510.4, out-of-sample deviance = 6567.7 ± 230.9 SE). The comparison of out-of-sample deviance for the three models indicates that the factor PTA contributed much to the prediction, but that there is a small additional value in adding age as a predictor (see Table 1), consistent with the weight ratio measured above.

As a second step, we investigated a fourth model (“PTA-based full model”) including not only main effects but also all interactions between predictors PTA, age, and cond. The resulting posterior estimates of the parameter weights were very close to those provided by the main-effect model, with very limited contributions from interaction terms (see the posterior distributions for all parameters in Supplementary Figure 2). The strongest interaction effect was obtained for *age* * *cond* (mean = 0.12). Although it was slightly different from zero (*CI*_95%_ = [0.03, 0.21], 2.6 SE away from zero), it was 11 times weaker than the main effect of PTA and 2.4 times weaker than the main effect of age. As before, these observations on model weights were confirmed by comparing the predictive accuracy of full- and main-effect models. The full model reached $\rho_{quiet}^{2}=47.7\%$ and $\rho_{noise}^{2}=59.6\%$ of variance explained, with D = 6286.4 and LOOIC = 6365.9 ± 235.4 SE out-of-sample deviance. It was therefore virtually indistinguishable from the main-effect model from the LOOIC point of view (see Table 1), indicating that interaction effects add no useful information for the prediction.

The negligible interaction effects should not be interpreted as evidence of independence between the different predictors, however, as they correspond to the logistic part of the model only (Equation 4). Even in the main-effect model including no explicit interaction terms, the slopes of the sigmoid function are different between the two conditions due to the multiplicative effect of *p*_*miss*_ (as can be seen from Figure 4). The maximum of the derivative of the model function respective to PTA was lower in quiet (−1.28 percentage point/dB HL) than in noise (−1.05 percentage point/dB HL, credible interval of the difference: *CI*_95%_ = [−0.28, −0.19]).

In an attempt to estimate the influence of audibility on speech identification scores, a fifth model was fitted on the HI data, replacing the logistic-linear effect of PTA on scores with a more realistic and empirically-grounded predictor of speech intelligibility, the ESII. All main and interaction effects were included. The predictive accuracy and posterior distributions for the ESII-based full model are reported in Table 1 and in Supplementary Figure 2, respectively. The out-of-sample predictive accuracy for this model was not reliably different from that of the PTA-based full model (nor from the PTA-based main-effect model). However, both models achieve this level of accuracy in a slightly different way, as revealed by the posterior distributions. On the one hand, contrary to the PTA-based model, the ESII-based model put almost no weight on factor condition (mean = 0.06, *CI*_95%_ = [−0.35, 0.44], 0.3 SE away from zero). This was to be expected as, in this case, the influence of noise was accounted for by ESII. On the other hand, while the interaction effect *PTA* * *cond* was negligible, *ESII* * *cond* was associated with a rather large coefficient (mean = 1.28, *CI*_95%_ = [0.98, 1.58], 8.4 SE away from zero).

**Figure 5:**
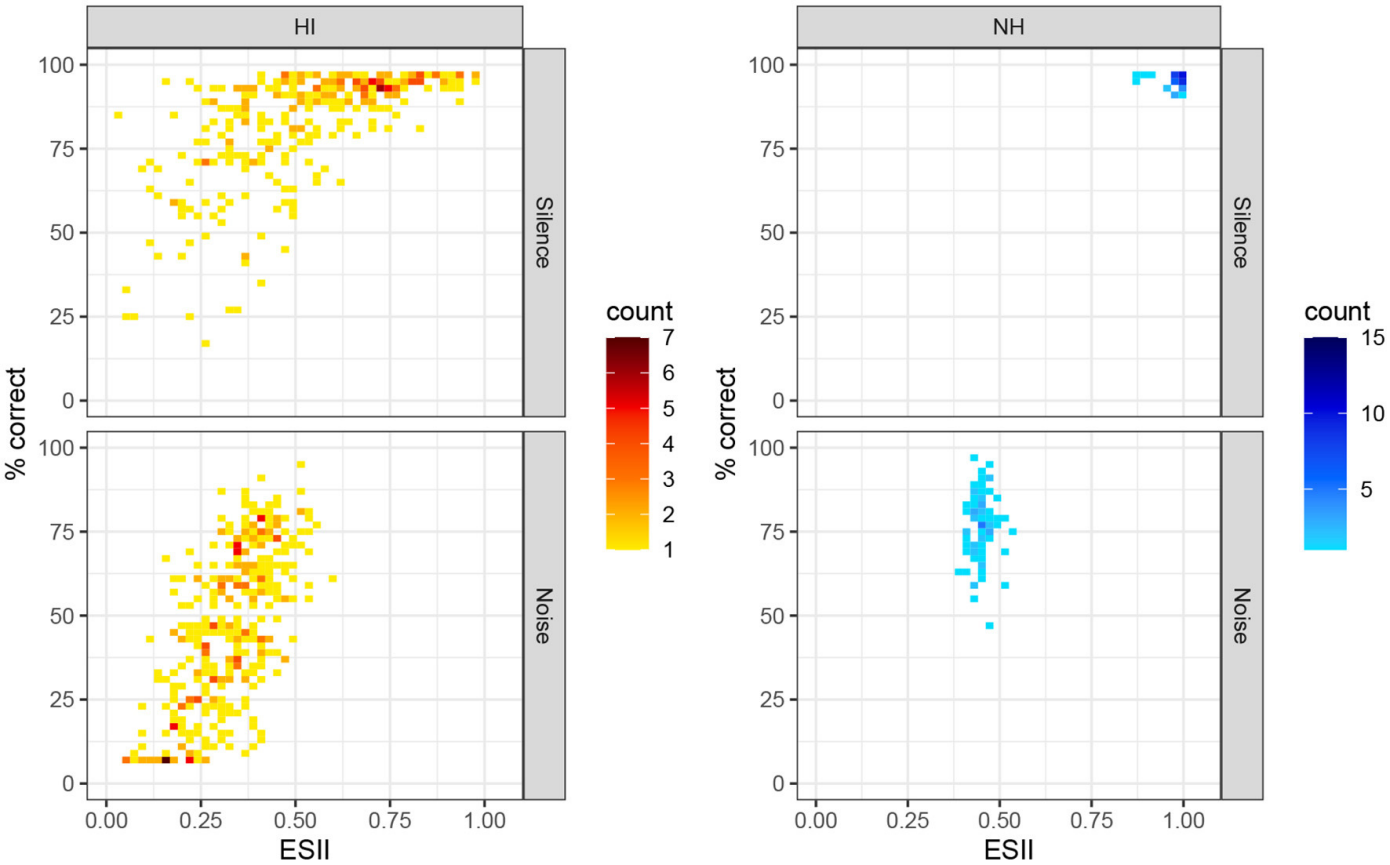
Intellitest scores in quiet and in noise for the two groups, as a function of ESII. Color scales indicate the total number of participants.

Finally, we turned to the comparison of variance in scores and ESII between HI and NH participants. On average, ESII values were lower in the HI group (mean = 0.57 ± 0.23 sd in quiet; 0.32 ± 0.11 in noise) than in the NH group (mean = 0.98 ± 0.03 sd in quiet; 0.45 ± 0.03 sd in noise), as can be seen in Figure 5. An ESII-based model including an additional effect of group was fitted on the full dataset. We computed the prediction errors of the model on the log-odds scale and compared their standard error between groups, as a measure of the dispersion of the data points around the regression curve. The posterior distributions for the standard deviation of prediction errors in the noise condition were 0.60 (*CI*_95%_ = [0.55, 0.69]) for NH and 0.80 (*CI*_95%_ = [0.79, 0.81]) for HI, indicating a larger variance of the scores left unexplained by the model in the HI group. As a validation, we computed a closely-related measure of the inter-individual variability of speech-in-noise performance within the two groups: the standard deviation of the scores on the log-odds scale for the NH group and for a subset of the HI group restricted to a “normal” range of ESII values (ESII ∈[0.35, 0.55]). Consistently with the previous measure, the standard deviation for the NH group was 0.67 for the NH group and 0.88 for the subset of the HI group.

## 4. Discussion

In the present study, we analyzed a dataset from a larger project in order to estimate the relative importance of PTA-related and age-related deficits in intelligibility loss in quiet and in noise. One strength of the dataset was the size and quality of the sample: 384 HI listeners, representative of the statistical distribution of absolute thresholds as a function of age and gender in France (AFNOR, 2017), recruited from audiological/ENT clinics in 7 French cities. Their performance in an aided phoneme-identification task in quiet and in a spectrotemporally modulated noise were entered into a Bayesian hierarchical model, allowing for a joint estimation of the effect of masking noise, PTA and age on intelligibility. Assuming that the DAG depicted in Figure 1 is correct, the weights associated to PTA, age, and cond in the full- and main-effect models can be seen as a measure of their direct causal effect on intelligibility.

As expected, the model including only main effects revealed that Intellitest scores were negatively associated with PTA, even though HI participants were provided with an individually-fitted frequency-dependent amplification. This is consistent with widely acknowledged explanations that (1) amplification cannot fully compensate for the audibility loss, and in some case even introduces additional distortions and that (2) while reduced audibility is the most visible part of sensorineural hearing loss, in most real-life situations, additional suprathreshold auditory deficits also come into play (Plomp, 1978; Pichora-Fuller and Souza, 2003; Lesica, 2018). The strength of the residual effect of PTA evidenced here may seem surprisingly high, however, with the slopes in Figure 4 reaching −1.28 percentage point/dB HL in quiet and −1.05 percentage point/dB HL in noise. Also informative in this respect is the amount of variance in speech-in-noise scores explained by PTA once age is controlled for ($\Delta \rho_{noise}^{2}$ between main-effect and age-only models = 28.2%, see Table 1). As noted in Introduction, it is not unusual that PTA contributes substantially to the variance in aided intelligibility. For example, Meister et al. (2015) showed that the mean audiometric threshold was the only significant predictor of hearing-aid users' performance in a monosyllabic words recognition test in quiet (*R*^2^≈ 54%) and in temporally-modulated speech-shaped noise (*R*^2^≈ 29%). Although these values cannot be compared directly with our *ρ*^2^ as they depend on the range of PTA tested, the precision of the outcome measure and the type of model used, they indicate that strong associations between PTA and aided intelligibility are to be expected in such context-free speech comprehension tasks.

In the present study, residual audibility loss is likely to play a role in the performance of HI listeners with the most severe loss in sensitivity, therefore potentially limiting the generalizability of the above findings. As illustrated in Figure 3.3,4 for the “mid-PTA” and “high-PTA” groups, the amplification provided by the Cambridge formula is insufficient to make the speech information above 2 kHz audible again. It is therefore plausible that the strong influence of PTA in the model reflects the effect of residual audibility loss for the participants with the most profound hearing impairment, particularly given the critical role of the frequencies above 2 kHz for non-sense-syllable recognition. For example, in Humes et al. (2013) the amplification used was spectral shaping individually to ensure optimal SII-based audibility up to 4 kHz for each listener. When doing so, aided speech perception was determined primarily by cognitive and higher-level auditory processes with a significant, but small, contribution from PTA. Another non-mutually exclusive explanation for the large β_*PTA*_ observed would be that suprathreshold deficits correlated with PTA severely impeded successful phoneme identification. In the absence of complementary auditory tests, it is difficult, however, to disentangle between the two possibilities. In an attempt to account for these potential limiting factors, we replaced the crude “PTA predictor” (which relies on the questionable assumption that intelligibility is governed by the average of 4 pure-tone thresholds) with a more realistic ESII predictor. In contrast to the PTA, ESII exploits the whole audiogram in relation with the stimulus content, and takes into account forward-making and certain aspects of temporal auditory processing (see section 2). Failing to account for individual differences on the basis of the ESII alone would indicate that other deficits contribute to speech-in-noise recognition. As a matter of fact, the ESII model yielded no better out-of-sample prediction accuracy as a PTA model with the same structure (Table 1). This suggests that the PTA model considered so far, although simplistic, accounted for the possible residual audibility loss and for the temporal processing deficits as well as the ESII model. Conversely, the variability in HI performance left unexplained by both models is likely to result from higher auditory or cognitive deficits, rather than from insufficient audibility.

Although both PTA and age were associated with strictly negative weights in the model, indicating that they jointly contributed to predicting the Intellitest scores, the effect of PTA was five times higher than the effect of age. This marginal role of age was confirmed by the comparison between main-effect and PTA-only models: adding variable age to the PTA-only model yielded a slight but consistent improvement in out-of-sample prediction accuracy (Table 1). Age-related intelligibility loss are often assumed to reflect primarily the deficit of cognitive functions such as short-term and working memory or attention skills (Meister et al., 2013; Füllgrabe et al., 2014). As already highlighted in the Introduction, researchers investigating the role of cognition in speech perception have found only modest effects, secondary to those of hearing loss, consistent with the previous study (Akeroyd, 2008). Furthermore, cognition is usually found to be a limiting factor only in the most demanding conditions, which require the listener to supplement the degraded acoustic input with top-down cognitive processing (Heinrich et al., 2015, 2016). For example, Heinrich et al. (2015) showed that an aggregated cognitive variable (including working memory, IQ and attention tests) was predictive for performance differences in sentence-in-modulated-noise recognition, but not in phoneme-in-steady-noise identification tasks. Similarly, the importance of cognitive factors in speech-in-noise test prediction crucially depends on the complexity of the masker (Schoof and Rosen, 2014; Nuesse et al., 2018). In particular, in Schoof and Rosen (2014), age-related deficits in speech perception were observed only in the presence of a two-talkers babble but not in steady or modulated noise. In the present study, the Intellitest consisted in a non-spatialized comprehension task on meaningless logatomes in non-linguistic noise or in quiet, therefore limiting the extent of possible cognitive compensatory processes recruited by HI participants. Therefore, the task was not the best suited to reveal an effect of cognition on speech perception, possibly explaining the marginal role of age in our model.

Another group of factors mediating the effect of age on intelligibility is auditory spectro-temporal processing skills. The spectro-temporally modulated noise condition included in the Intellitest is likely to entail high-level auditory mechanisms, such as “listening into the dips,” the ability to catch brief acoustic glimpses of speech when fluctuating background noise levels momentarily drop (Peters et al., 1998; Bronkhorst, 2000; Cooke, 2006). For consonant identification tasks, the release from masking provided by the presence of temporal fluctuations in the masker has been estimated to approximately 25 percentage points for NH listeners (Füllgrabe et al., 2006; Lorenzi et al., 2006; Phatak and Grant, 2012) and ~ 10 percentage points for HI listeners (Lorenzi et al., 2006). As additional processes are engaged in the presence of a fluctuating background, the relative importance of sensitivity and suprathreshold and cognitive processes are modified. For example, George et al. (2007) and Van Esch and Dreschler (2015) reported that the pure-tone audiogram was the predominant factor when predicting HI listeners SRT for sentences in stationary noise, but that other (auditory and cognitive) factors contributed to explaining interindividual differences when temporal fluctuations were introduced in the masker. Unfortunately, in the absence of a steady-noise condition in the Presbycusis project data, we were not able to disentangle the specific contribution of modulation masking-release from other auditory mechanisms.

We then turned to a more complex model including all interaction terms between factors PTA, cond and age. As argued in the previous paragraphs, compared to speech-in-quiet comprehension, speech-in-noise comprehension entails additional, higher-level processes which can be impacted by aging and hearing loss. We therefore anticipated that the relative importance of PTA and age depended on condition, resulting in non-null interactions *cond* * *PTA* and *cond* * *age*. Surprisingly, however, the posterior distributions obtained for the PTA-based full model indicated that interaction effects, if any, were more than 11 times weaker than the main effect of PTA. As a consequence, the contribution of these effects to prediction accuracy was negligible: the full model was virtually indistinguishable from the main-effect model from the LOOIC point of view (Table 1). Nevertheless, it should be reminded that, in our model, the effect of noise is two-fold: a logistic-additive effect of the factor cond and a multiplicative effect through *p*_*miss*_. The latter is usually thought of as a baseline maximum correct response rate; yet, it also influences the predicted scores on the whole range of PTA and age. As a result, even in the main-effect model, the PTA slopes differ between conditions, indicating that the presence of modulated noise lowered the influence of PTA on scores, as predicted from the literature. Although real, this effect on the slopes was limited in size (−1.28 percentage point/dB HL in quiet vs. −1.05 percentage point/dB HL in noise) maybe because of the non-linguistic nature of the masker.

As a last step, we sought to compare the NH and HI groups on the basis of the individual variance in performance not explained by audibility. For this purpose, we fed the complete dataset, including HI and NH scores, to the ESII-based model, then measured the unexplained variance for the two groups after accounting for audibility. There was a non-negligible variability in the NH scores for the noisy condition (sd = 10 percentage points, 0.67 on the log-odds scale) despite very little heterogeneity in ESII (mean = 0.45 ± 0.03). As a consequence, the standard deviation of the residual of the ESII model on the NH group was very close to the standard deviation of their scores (sd of residuals on the log-odds scale = 0.6). Conversely, the HI group covered a broader range of speech-in-noise scores and ESII values (from 0 to 0.6). The dispersion in the performances after accounting for audibility was again estimated as the standard deviation of the residuals of the ESII model. The HI data points were more widely dispersed around the regression curve than the NH scores (sd of residuals on the log-odds scale = 0.8). In a related way, a subset of the HI group matched with the NH group in ESII values yielded a standard deviation of scores of 19 percentage points (sd = 0.88 on the log-odds scale). Figure 5 provides a sense of the dispersion of individual scores for the two groups. While the variance in speech-in-noise performance was large for the NH and HI groups, even after accounting for audibility and age (as well as a part of the temporal processing deficits) with the ESII model, wider heterogeneity in the HI group may reflect the contribution of higher-level suprathreshold deficits, such as a suboptimal use of information in the central auditory system (Varnet et al., 2019; Venezia et al., 2019).

A strength of the Presbycusis project dataset is the large size of the sample and the set of inclusion criteria chosen so as to make it representative of the French population. However, because the data was collected as part of a separate project, one limitation of the present analysis is the limited number of relevant variables measured for each individual. In particular, the lack of low-level auditory tests made it impossible to disentangle between the different suprathreshold deficits and cognitive factors associated to aging. Similarly, as noted above, we were not able to estimate the specific contribution of modulation-masking release, due to the absence of a steady-noise condition. Another type of explanatory variable which is worth considering for causal inference analysis of presbycusis is genetic factors. A genome analysis was performed for all HI recruited within the present project. The comparison with phenotypic variance demonstrated that the genetics of presbycusis is shaped not only by well-studied polygenic risk factors of small effect size revealed by common variants but also by ultrarare variants (Boucher et al., 2020). Further efforts should be devoted in the future to expand the DAG in Figure 1 by including whole-exome sequencing data as well as complementary auditory tests, to draw more detailed conclusions on the relative contributions of age, audibility, and genetic factors to speech intelligibility deficits in presbycusis.

## Data Availability Statement

Publicly available datasets were analyzed in this study. This data can be found here: https://github.com/LeoVarnet/Presbycusis_analysis.

## Ethics Statement

The studies involving human participants were reviewed and approved by French Regional Ethics Committees CCPPRB Paris Necker and CPP Ile de France II. The patients/participants provided their written informed consent to participate in this study.

## Author Contributions

CL, CP, and AL conceived and planned the experiments. LV carried out the statistical analysis of the data. AL computed the Extended Speech Intelligibility Index. All authors contributed to the interpretation of the results. LV wrote the manuscript with contributions from all authors. SB and CB contributed to the analysis of clinical records.

## Conflict of Interest

The authors declare that the research was conducted in the absence of any commercial or financial relationships that could be construed as a potential conflict of interest.

## Footnotes

^1^Note that percentages of explained variance (*R*^2^) depend on the precision of the tests relative to the range of PTA included in a study, as well as the exact task and condition. Still, we found it interesting to report their orders of magnitude here to illustrate the strength of the dependencies between the variables of interest.

^2^Age can also have an indirect positive influence on speech comprehension for hearing-aid users, as individuals with a relatively stable hearing-loss pattern are able to adapt their listening strategies to optimally decode the impoverished speech signals that they receive (Seldran et al., 2011). Nevertheless, given the overall negative association of age observed in all the studies cited above, this effect can be regarded as negligible and will not be further considered.

^3^Note that these counterfactual predictions, showing the effect of varying PTA independently of all other parameters, slightly underestimate the dependency between PTA and scores in the measured data. This was indeed expected from the relationship between PTA and age: participants with a high PTA are likely to be older, and therefore to have other deficits. For a visual illustration of the goodness of fit of the model, a complementary figure showing the expected scores according to the model is provided as supplement to this article (Supplementary Figure 3).
