# Supplementary material for: Contributions of Age-Related and Audibility-Related Deficits to Aided Consonant Identification in Presbycusis: A Causal-Inference Analysis
Source: Front Aging Neurosci. 2021 Mar 1;13:640522. doi: 10.3389/fnagi.2021.640522 (PMC7956988; doi:10.3389/fnagi.2021.640522)
Supplement: Supplementary file 1 [file Presentation_1.PDF]

Supplementary Material

Supplementary material for the article "Contributions of age-related and audibility-related deficits to aided consonant identification in presbycusis: a causal-inference analysis" (L. Varnet, A. Léger, S. Boucher, C. Bonnet, C. Petit, C. Lorenzi, Frontiers in Aging Neuroscience, 2021).
Correspondence: leo.varnet@ens.psl.eu

1 MODEL DESCRIPTION

Five models were investigated in this study. They differ only in the parameters included in the standard logistic regression formula (equation 4). The initial model ("PTA-based main-effect model") includes only the main effects of PTA, age, cond and gender:

$$logit(p') = \gamma_{0,site} + \beta_{PTA} \cdot PTA + \beta_{age} \cdot age + \beta_{cond} \cdot cond + \beta_{gender} \cdot gender$$

where PTA and age are standardized vectors. The hierarchy of priors associated with each parameter in the model is summarized in figure S1.

Two nested models (age-only model and PTA-only model) are derived from the above equation by removing factors PTA and age, respectively – all other things being equal:

$$logit(p') = \gamma_{0,site} + \beta_{age} \cdot age + \beta_{cond} \cdot cond + \beta_{gender} \cdot gender$$

$$logit(p') = \gamma_{0,site} + \beta_{PTA} \cdot PTA + \beta_{cond} \cdot cond + \beta_{gender} \cdot gender$$

The PTA-based full model includes all interaction effects between factors age, $cond$ and PTA:

$$logit(p') = \gamma_{0,site} + \beta_{PTA} \cdot PTA + \beta_{age} \cdot age + \beta_{cond} \cdot cond + \beta_{gender} \cdot gender + \beta_{cond*PTA} \cdot PTA \cdot cond + \beta_{age*cond} \cdot age \cdot cond + \beta_{age*PTA} \cdot age \cdot PTA + \beta_{age*cond*PTA} \cdot age \cdot PTA \cdot cond$$

Finally, the ESII-based full model replaces the factor PTA in the previous equation with a more perceptually plausible index of audibility, the ESII:

$$logit(p') = \gamma_{0,site} + \beta_{ESII} \cdot ESII + \beta_{age} \cdot age + \beta_{cond} \cdot cond + \beta_{gender} \cdot gender + \beta_{cond*ESII} \cdot ESII \cdot cond + \beta_{age*cond} \cdot age \cdot cond + \beta_{age*ESII} \cdot age \cdot ESII + \beta_{age*cond*ESII} \cdot age \cdot ESII \cdot cond$$

The posterior distribution of each parameter in the 5 models are represented in figure S2. The expected scores (individual posterior predictions in the two condition) for the PTA-based main-effect model are shown in figure S3, together with the real data. Finally, figure S4 provides an insight into the variability of the scores across testing sites as well as into the role of the site-specific $\gamma_{0,site}$ intercept parameters in the PTA-based main-effect model.

2 SUPPLEMENTARY FIGURES

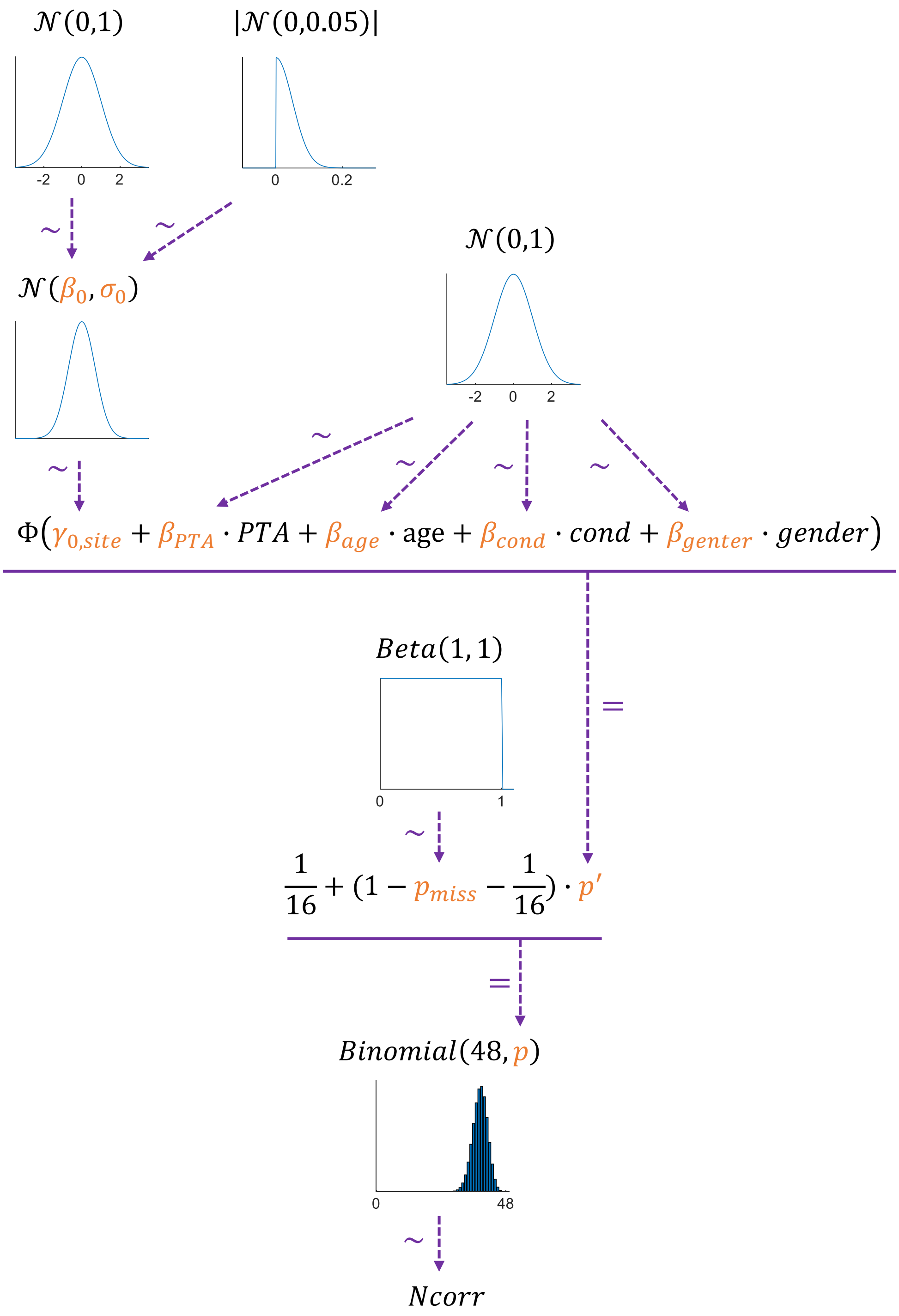

Figure S1. Schematic diagram of the main Bayesian hierarchical model used in this study ("PTA-based main-effect model"). The chain of arrows illustrates the chain of dependencies between parameters and prior distributions. Arrows with the sign $\sim$ denote stochastic dependencies, while arrows with the sign $=$ represent deterministic dependencies. Parameters estimated within the model are indicated in orange. Φ denotes the logistic function.

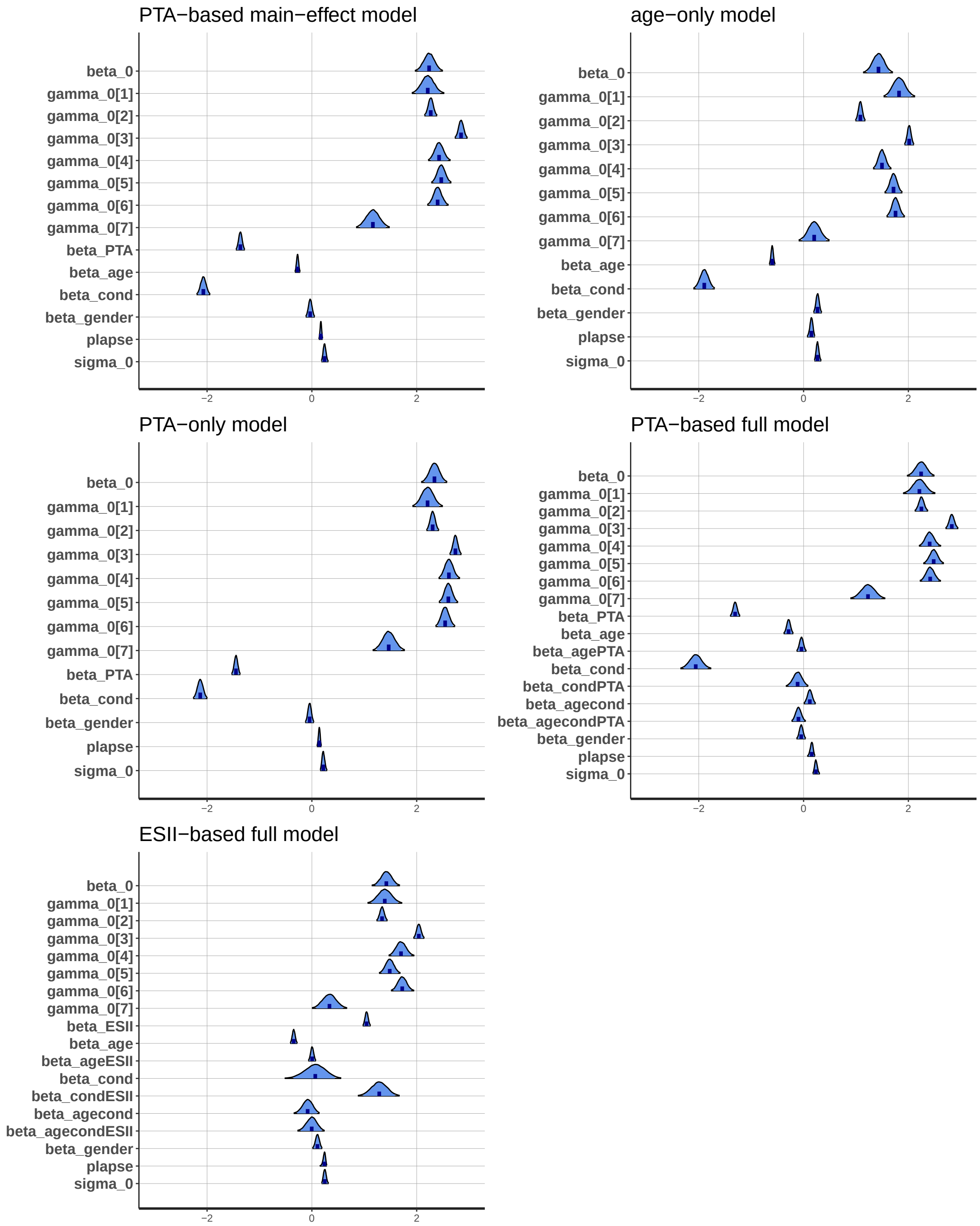

Figure S2. Posterior distributions of all parameters for each model considered in this study. The 95% credible interval for each estimate is indicated in blue.

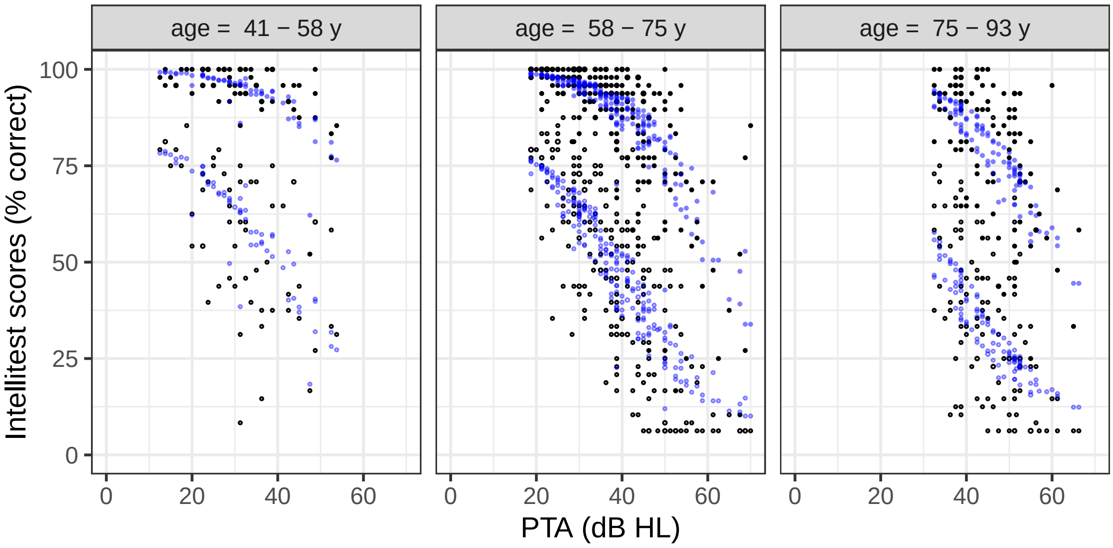

Figure S3. Intellitest scores in quiet- (solid black dots) and noise- (open black dots) conditions for the HI group, as a function of PTA and age (same representation as in Figure 4A). Blue dots correspond to the expected scores for each participant in each condition, according to the PTA-based main-effect model.

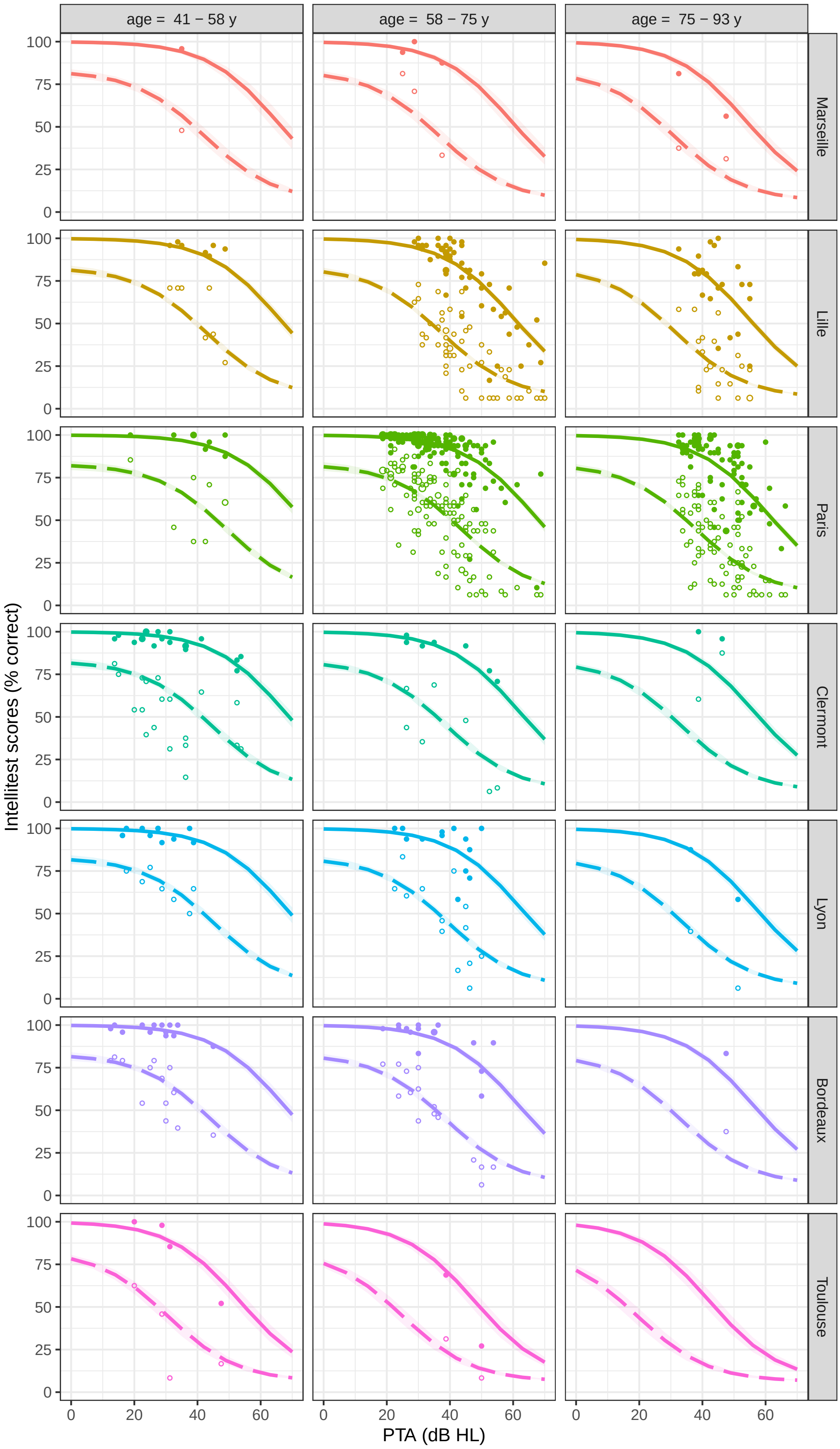

Figure S4. Intellitest scores in quiet- (solid dots) and noise- (open dots) conditions for the HI group, as a function of PTA and age (same representation as in Figure 4A). The different colors show the data from each testing site as well as site-specific predictions (i.e. using the site-specific intercepts $\gamma_{0,site}$).
